# Supplementary material for: Are carbon emissions trading and green financial instruments synergistic? -Comprehensive quantitative research based on content analysis
Source: PLoS One. 2024 Mar 7;19(3):e0298601. doi: 10.1371/journal.pone.0298601 (PMC10919601; doi:10.1371/journal.pone.0298601)
Supplement: S1 Data — (ZIP) [file pone.0298601.s001.zip › Data/地级市Z_F工作报告.zip!/╣π╬≈/└┤▒÷╩╨/└┤╫╘╣1⁄2╓┌║┼í╛┬φ┐╦╩2╛▌═°í┐_2016.docx]

各位代表：

现在，我代表市人民政府向大会报告工作，请各位代表连同《来宾市国民经济和社会发展第十三个五年规划纲要（草案）》一并审议，并请各位政协委员和其他列席会议的同志提出意见。

一、2015年和“十二五”工作回顾

2015年，在自治区党委、政府和市委的正确领导下，全市各级政府和各族人民坚持以邓小平理论、“三个代表”重要思想和科学发展观为指导，认真贯彻落实习近平总书记系列重要讲话精神，面对建市以来最为严峻的形势，我们认真贯彻落实自治区稳增长“48条”，出台我市稳增长“49条”，及时应对和解决遇到的突出困难和问题，着力扭转经济下滑态势，保持经济社会平稳发展。初步统计，全市实现地区生产总值557.70亿元，增长3.4%；固定资产投资449.07亿元，增长4.1%；财政收入50.02亿元，非税收入占比同比下降7.6个百分点；社会消费品零售总额145.11亿元，增长8.2%；城镇居民人均可支配收入27077元，增长6.6%；农民人均纯收入8379元，增长8.1%。

“十二五”时期，我们坚持“环境立市、工业强市、城建塑市、农业稳市、商贸旺市”发展战略，坚持走“调整型、追赶型、特色型、绿色型”可持续发展之路，克难攻坚，扎实工作，全市经济社会平稳发展，为同步全面建成小康社会打下坚实基础。

——过去五年，我们励精图治、共克时艰，全市经济发展取得新成效。面对严峻的宏观经济发展形势，我们妥善应对各种风险挑战，经济综合实力进一步提升。地区生产总值由2010年的405.22亿元增加到557.70亿元，年均增长7.4%；三次产业结构由2010年的24.1：47.5：28.4调整为24.5：39.1：36.4；财政收入由43.05亿元增加到50.02亿元，年均增长3.0%；金融机构人民币贷款余额由186.70亿元增加到358.50亿元，增长92.0%。

——过去五年，我们与时俱进、改革创新，全市经济社会发展迸发新活力。市本级行政审批事项从368项缩减到148项，减少60%。完成市、县两级政府部门权力清单及责任清单的编制和公布工作。新增私营企业8029户，比“十一五”时期增长135.7%。全面完成市县两级政府职能转变和机构改革。财政金融体制改革有效推进，共化解政府性债务269.57亿元。国资和国有投融资企业、农村金融服务体系、农村土地制度、县级公立医院、公共资源交易体制、户籍制度、公务用车制度等改革取得良好成效。

——过去五年，我们主攻园区、力促转型，工业发展转型升级迈出新步伐。累计投入60亿元建设园区基础设施，全市自治区级产业园区达8个，实现6个县（市、区）自治区级产业园区全覆盖。来宾高新技术产业开发区成功升格为自治区级重点产业园区。茧丝绸、碳酸钙、汽车（机械）配件等新兴产业发展势头良好。铝精深加工基地成为自治区重点打造的十五个产业基地之一，热电联产规模和品质居全区首位。新增科技企业孵化器2家。

——过去五年，我们调优结构、打造品牌，现代农业发展取得新成果。农林牧渔业总产值年均增长4.5%。农业机械化水平达50.3%，进入中级发展阶段。粮食产量平稳增长，桑蚕、水果、肉牛、水产品等特色农业发展迅速。落实糖料蔗“双高”基地建设10.53万亩，甘蔗单产提高10.4%。获得国家、自治区级“三品一标”认证农产品53个。启动创建市级以上现代特色农业（核心）示范区15个，其中来宾高新区金凤凰果蔬产业基地成功创建自治区级示范区。重点龙头企业54家，家庭农场246家，专业合作社855家。农村土地确权登记颁证工作成为全国试点。

——过去五年，我们优先旅游、强化商贸，三产发展取得新突破。社会消费品零售总额年均增长12.8%。旅游产业发展迅速，实现各县（市、区）A级旅游景区全覆盖，金秀县创建广西特色旅游名县通过评定，忻城县进入备选县行列。累计接待游客5065万人次，实现旅游收入293.0亿元，分别是“十一五”的 2.9倍和5.3倍。沃尔玛、国美电器、肯德基等知名企业相继进驻，城区建成桂中农贸综合批发市场、果蔬批发市场等一批专业市场。累计销售商品房661.01万平方米，年均增长12.8%。金融、信息、拍卖、二手车交易、家政等中介服务快速发展。

——过去五年，我们扩大投资、补齐短板，基础设施建设实现新跨越。固定资产投资年均增长17.8%。桂中治旱一期工程完成投资18.25亿元，占概算总投资68.9%，二期工程开工建设。桂中农村土地整治重大工程建成高标准农田153.87万亩，小型水利渠道5630公里。新增铁路86.5公里，高铁开通运营，来宾迈进高铁时代。新增高速公路175公里，来宾至武宣、来宾至马山、柳州至武宣高速公路建成通车。新增港口吞吐能力383万吨，总吞吐量突破5000万吨，来宾宾港作业区二期工程、象州港区猛山作业区建成运营，红水河内河航线断航38年后复航。供水、供电、供气、通讯、污水垃圾处理等基础设施日益完善。

——过去五年，我们科学规划、精心打造，新型城镇化建设展现新面貌。主城区建成面积从“十一五”末的31平方公里扩大到43平方公里，预计全市城镇化率41%，年均提高1.49个百分点。相继建成三桥、四桥等20多座城市桥梁，28公里内环建成通车。着力打造山水园林宜居城市，城区绿化覆盖率达39.1%，荣获“广西园林城市”称号，“桂中水城”魅力初显。获列为国家新型城镇化综合试点城市，兴宾城南新区、象州象东新区、武宣城东新区、忻城城南新区、金秀县城民族化改造、合山国家矿山公园、来宾高新区展现新面貌，石龙镇、桐木镇、红渡镇等城镇建设成效显著。实施城市网格化管理，大数据建设稳步推进。

——过去五年，我们差别考核、建治并重，生态文明建设取得新进展。森林覆盖率达51.35%，成功创建“广西森林城市”。万元GDP能耗下降13%以上，万元工业增加值能耗下降15.1%，均提前完成“十二五”目标任务。市城区生活垃圾无害化处理率达100%，污水处理率85%以上。全面开展“美丽来宾”乡村建设活动，农村生态和人居环境质量明显提升。全市环境质量保持良好，各类指标均达到或好于有关标准。

——过去五年，我们统筹兼顾、协调推进，县域经济发展呈现新景象。兴宾区突出发展工业“园中园”经济，初步建成制糖及综合利用、家具制造等产业体系。象州县力推园区、港口、仓储基础设施建设，茧丝绸、电动车、汽配等产业不断壮大。武宣县重点打造矿产品精深加工基地和区域物流中心，港口物流、铅锌矿加工、碳酸钙等产业发展迅速。忻城县加强“一区四园”建设，形成以石材、桑蚕等为主体的产业发展格局。金秀县全力打造“生态、民族、长寿”三大品牌，初步形成旅游、瑶医瑶药、绿色生态产品等养生健康产业体系。合山市加快推进工业转型，电力、碳酸钙、旅游等产业不断发展壮大。来宾高新区重抓园城同建、产城一体化建设，信息技术、新材料、先进制造等产业加快发展。

——过去五年，我们兜住底线、增进福祉，民生事业发展取得新进步。城镇居民人均可支配收入年均增长9.3%，农民人均纯收入年均增长12.5%。新增城镇就业9.60万人、农村劳动力转移就业30.30万人。养老、基本医疗等五项保险参保人数78.10万人次，城乡居民享受最低生活保障人数166.27万人次。投入救灾资金 6624.10万元，救助群众64.90万人次。保障性安居工程建成入住2.75万套。完成农村危房改造7.56万户。27.5万人实现脱贫。解决农村饮水不安全人口97.31万人。投入3.49亿元，坚持每年办好10件实事。累计投入100亿元，完善教育基础设施。全市学前三年毛入园率达75%，居全区前列。义务教育均衡发展水平居全区首位。6个县（市、区）实现自治区示范性高中全覆盖。职业教育改革稳步推进。柳州师专升格为广西科技师范学院，本科层次高等教育实现历史性突破。成功创建自治区卫生城市。新农合参合率连续五年超额完成目标任务。建成“国家公共文化服务体系示范区” 和“全国全民健身示范城市”。成功创建自治区级文明城市。获得专利授权636件，是“十一五”的5.3倍。

社会治理创新、治安管控、信访、人民调解和行政调解、公共法律服务体系建设、产品质量、食品药品安全等不断加强，群众安全感满意度排名稳步提升，2015年排名全区第2。“全国双拥模范城” 创建工作稳步推进，军政军民关系更加密切融合。民族宗教、外事侨务、人防、消防、统计、水文、气象、水库移民、供销、社会科学、政策咨询、地方志、档案、工会、共青团、妇女儿童、老龄、残疾人等工作取得新进步，银行、税务、电力、烟草、通信、盐务等为地方发展作出了积极贡献。

五年来，我们加快转变政府职能，依法行政和依法治市扎实推进。认真执行市人大及其常委会决定，自觉接受市政协民主监督。人大代表议案和政协委员提案办结率100%。认真贯彻落实中央八项规定精神，扎实开展党的群众路线教育实践活动和“三严三实”专题教育，廉政建设和反腐败斗争取得明显成效。

回顾五年来的工作，发展的历程极不平凡，取得的成绩实属不易。这是自治区党委、政府和市委正确领导的结果，是市人大依法监督、市政协民主监督的结果，是各级各部门、全市各族人民同心协力、艰苦奋斗的结果，是社会各界热情关心、大力支持的结果。在此，我代表市人民政府，向全市各族人民，向人大代表、政协委员，向所有关心和支持来宾发展的各界人士，表示崇高的敬意和衷心的感谢！

在总结成绩的同时，我们也清醒地看到前进中的困难和挑战。主要是经济基础仍然较为薄弱，总量小，经济下行压力大；工业化水平不高，传统产业增长动力衰减，转型升级难度大，新的增长点不多；农业现代化水平不高，产业竞争力弱，提质增效难度大；城镇化发展水平较低；城乡居民收入不高，扶贫攻坚任务还很艰巨；社会管理和服务水平有待提高。等等。对这些问题，我们将高度重视，认真研究，攻克难关，切实加以解决。

二、“十三五”时期经济社会发展主要目标和任务

按照市委三届八次全会精神，我市“十三五”时期发展的总体要求是：高举中国特色社会主义伟大旗帜，以马克思列宁主义、毛泽东思想、邓小平理论、“三个代表”重要思想、科学发展观为指导，深入贯彻落实习近平总书记系列重要讲话精神，全面贯彻党的十八大和十八届三中、四中、五中全会精神，坚持“四个全面”战略布局和“五位一体”总体部署，牢固树立创新、协调、绿色、开放、共享发展理念，围绕全面建成小康社会目标，突出科学发展主题，以打造来宾经济升级版为主线，按照稳中求进工作总基调，深入实施“环境立市、工业强市、城建塑市、农业稳市、商贸旺市、依法治市”发展战略，把来宾打造成为广西“双核驱动”战略重要节点城市。

根据《中共来宾市委关于制定国民经济和社会发展第十三个五年规划的建议》，市政府组织编制了“十三五”规划《纲要（草案）》。《纲要（草案）》提出“十三五”时期经济社会发展的目标是：

——经济持续较快发展。地区生产总值年均增长8%，比2010年翻一番以上；财政收入年均增长6%；固定资产投资年均增长12%；社会消费品零售总额年均增长8%；价格总体水平保持稳定。经济增长质量和效益提高。

——人民生活水平和质量全面提高。居民人均可支配收入年均增长8%，比2010年翻一番以上。新增城镇就业4万人。现行标准下农村贫困人口全部脱贫，贫困县、贫困村全部“摘帽”。

——基本公共服务均等化总体实现。深化教育综合改革，新增劳动力平均受教育年限达13年，普及15年基本教育，与全区同步基本实现教育现代化。社会保障全民覆盖，人人享有基本医疗卫生服务，住房保障体系基本形成。国家公共文化服务体系示范区和全国全民健身示范城市进一步巩固和提升。全国文明城市创建工作顺利完成。公共法律服务体系基本建成。

——改革开放全面深化。政府职能进一步转变，重要领域和关键领域改革取得明显成效。基本建成珠江—西江经济带重要节点，形成开放型经济体系。

——结构调整取得重大突破。供给侧结构性改革取得进展，农业根基稳固，工业新兴产业发展成为全市经济的重要支撑，服务业增加值占地区生产总值比重提高3个百分点。研究与试验发展经费支出占地区生产总值比重达到1%。

——新型城镇化综合试点加快推进。到2020年，全市农业转移人口30万人，市主城区人口达到50万人，常住人口城镇化率达到50%。

——基本建成生态文明示范区。到2020年，主要生态环保指标达到国家和自治区要求，全面完成节能减排目标任务，森林覆盖率力争达到53%，城乡环境质量明显改善。

——民主法治健全完善。民主制度更加健全、形式更加丰富。全面依法治市，社会治理体系和治理能力现代化取得重大进展。

《纲要（草案）》提出“十三五”时期经济社会发展的主要任务措施是：

（一）实施创新驱动战略。围绕传统产业升级、新兴产业和现代农业发展，提高科技创新能力，推动科技和产业深度融合发展。搭建创新平台，推动大中型企业加快建立研发机构。加快来宾高新区建设，把高新技术园区培育成为我市创新驱动的前沿。

（二）构建全方位开放合作新格局。把握 “一带一路”建设和珠江—西江经济带开放开发的机遇，深化与粤港澳台地区及东盟国家的投资与交流合作。利用资源和区位优势，大力招商引资。

（三）打造现代工业产业体系。加快组建来宾市大工业专用区域电网，破解火电、冶金、制糖、铝加工等传统支柱产业用电瓶颈问题。到2020年，把电力、冶金、制糖、铝加工、农产品加工等支柱产业发展成为优势产业，把碳酸钙、茧丝绸、汽车（机械）配件等新兴产业培育为新支柱产业。

（四）加快发展现代农业。到2020年，全市粮食播种260万亩、总产量85万吨以上。加快推进优质稻、水果、桑蚕、生猪、肉牛等特色优势农产品产业基地建设步伐。加快现代特色农业（核心）示范区创建工作，每个县（市、区）建成自治区级1个以上、市级2~3个。建成糖料蔗“双高”基地105万亩。全面完成农村土地确权工作。农业综合机械化率72%以上。

（五）促进现代服务业提速增效。重点建设宾港现代物流、义乌（来宾）小商品、来宾高新科技园、金秀瑶都生态养生服务基地、城北金融等服务业集聚区。加快发展商贸物流业，构建覆盖城乡的电商平台。加快发展旅游业，到2020年，力争实现年接待游客3000万人次，旅游总收入250亿元。

（六）全力推进精准扶贫精准脱贫。深入实施“产业扶贫、基础设施建设、教育扶贫、扶贫培训、扶贫移民搬迁、金融扶贫、旅游扶贫、社会扶贫、结对帮扶、救济帮扶等十大扶贫工程”，实现27.3万贫困人口全部脱贫，忻城县、金秀县、武宣县和全市贫困村摘掉贫困“帽子”。

（七）推进城乡发展一体化。加快国家新型城镇化综合试点城市建设，让农民“想进来、进得来、留得住”，走出一条特色鲜明的新型城镇化道路。推进港产城融合发展，促进城乡一体化。

（八）建设完善基础设施。力争新改建高速公路431.8公里，一级公路144公里。以打造2000吨级内河港口为目标，加快港区建设。完善铁路运输通道。加快发展通用航空。加快推进水利设施建设。完善给排水、污水垃圾处理、道路等市政基础设施。

（九）优化空间发展格局。实施主体功能区战略，推动市县乡严格按照主体功能定位发展。以主体功能区为基础，推动经济社会、城乡建设、土地利用 “三规合一”，实现一张蓝图绘到底。

（十）创建绿色低碳生态文明示范区。加强土地、水、港口岸线等资源节约集约利用。严格源头预防，着力解决突出环境问题。积极发展生态工业、农业和服务业，因地制宜建设一批新型生态产业园。构建资源循环利用体系，推进低碳型社会建设。

（十一）建设共享幸福美好生活。实施更加积极的就业政策，稳定和扩大就业。深入实施教育振兴计划。完善城乡社会保障体系，着力解决社会保险、医疗卫生、住房等民生问题。拓展城乡居民收入渠道，创造条件增加财产性收入和政策性收入。

（十二）传承繁荣来宾文化。推动全民树立社会主义核心价值观。重点策划壮族和瑶族文化博览中心、兴宾区麒麟山壮族始祖文化园、忻城莫土司衙署建筑群落、武宣庄园系列、象州运江古镇、合山奇石文化村等一批文化产业项目。大力发展文化新闻出版广电事业，推进传统媒体与新媒体融合发展。

（十三）构建和谐包容的社会。全面推进依法治市，建设法治来宾。深化社会治安防控体系建设，着力提高社会安全感满意度。建立健全依法维权和调处化解矛盾纠纷综合机制。推进覆盖城乡的公共法律服务体系建设。深入贯彻落实党的民族政策，保障少数民族合法权益，巩固发展平等团结、互助和谐的民族关系。

各位代表！我市“十三五”发展的宏伟蓝图已经绘就，站在新的发展起点上，我们有信心和决心，带领全市人民应对挑战、破解难题，推动全市经济社会在科学发展的道路上阔步前进，开创更加美好的新生活！

三、2016年工作安排

2016年是全面建成小康社会决胜阶段的开局之年，也是推进结构性改革的攻坚之年。我们将认真贯彻落实创新、协调、绿色、开放、共享五大发展理念，全面贯彻落实市委三届八次全会精神，坚持走调整型、追赶型、特色型、绿色型发展之路，实施新兴产业培育壮大、传统产业二次创业、中小企业扶持成长、现代农业增量提质、服务业集聚提升“五大工程”，扎实开展工业提质、服务业提升、项目建设落实、西江经济带建设推进、精准脱贫攻坚“五个年”活动，奋力实现“十三五”良好开局。

全年经济社会发展的主要预期目标是：地区生产总值增长6%左右；财政收入增长6%左右；规上工业增加值增长7%左右；固定资产投资增长9%左右；社会消费品零售总额增长8%左右；居民人均可支配收入实际增长7.5%左右；城镇登记失业率控制在4.1%以内；常住人口城镇化率43%；农村贫困人口减少8.5万人；人口自然增长率控制在9‰以内；完成自治区下达的节能减排任务。

提出上述目标主要是坚持必要性、可能性和可行性的统一，充分考虑了实现地区生产总值2020年比2010年翻一番的需要和新常态下我市发展的各项支撑条件。完成今年的目标任务，要重点抓好以下工作：

（一）大力开展工业提质年活动，推进工业发展转型升级。

加快推进“工业强市”步伐，围绕打造先进制造业、热电联产循环经济、铝电一体化精深加工、碳酸钙产业、茧丝绸及服装加工等“五大基地”，加快工业转型升级。加快推进“五大工程”中的三大产业工程，把“工业提质年”真正转变为“信心提振年”。

抓好新兴产业培育壮大工程。认真落实《中国制造2025》和“互联网+”行动计划，重点培育碳酸钙、茧丝绸、高新技术、新能源、生物及民族医药等新兴产业和先进制造业，力争产值达到70亿元，其中碳酸钙、茧丝绸、汽车（机械）配件等三大产业产值突破40亿元。

抓好传统产业“二次创业”工程。加快工业电力体制改革，促进大工业专用区域电网建设。认真落实自治区糖、铝“二次创业”方案。扩大热电联产规模，建设覆盖河南工业园区和来宾高新区的供热管网，进一步延伸产业链。加大技术改造投入，引导企业深化对信息技术和智能制造的应用。

抓好中小企业扶持发展工程。完善企业培育上规工作机制，力争新增规上企业15家。大力推行“惠企贷”融资，力争完成信贷引导融资2亿元以上。

抓好重点工业项目建设。全力推动华润水泥（武宣）二期工程、福美软瓷新材料产业园等13个项目实现开工建设；加快推进河南工业区热电联产循环经济示范基地等29个续建项目建设；确保国鼎冰糖、桂诚纸业等9个项目实现竣工投产。

抓好工业园区建设。推进工业园区管理体制机制改革，确保改革措施落实到位。加快来宾高新区建设和红河工业集中区、象州县工业集中区、武宣县工业园区产城互动建设。扎实推进金秀、忻城扶贫产业园区建设。完善园区配套设施建设。

抓好服务企业活动。制定切实可行的服务方案，对重点企业实施“一企一策”，积极协调解决企业生产运行、项目推进中遇到的困难和问题，用真心真情真力全力扶持企业发展。

（二）大力实施现代农业增量提质工程，加快现代农业发展。

以建设现代特色农业（核心）示范区、调整农业产业结构、提高农业产品品质、提升农产品电子商务销售水平为引领，加快推进现代农业发展，力争农林牧渔业增加值增长3%。

加强农业基础设施建设。加快桂中治旱工程建设，一期完成投资5.5亿元，实现总干渠全面贯通，二期完成投资4亿元。完成整县推进高标准农田土地整治项目建设。继续抓好中央投资农田水利重点县水利工程建设，建成一批防洪、抗旱应急工程。继续推进下六甲灌区节水配套改造前期工作。

优化农业产业结构。坚持“稳面积、增单产”，确保粮食总产81.5万吨以上。全力推进27万亩糖料蔗“双高”基地建设。狠抓区市县乡四级现代特色农业（核心）示范区创建工作，力争创建自治区级示范区1个以上。积极发展水果、桑蚕、蔬菜、畜禽、水产品等特色高效农业。加大农产品深加工、食品工业项目的引进和建设力度。加快推进海升集团2500亩现代化柑橘种植基地、正大集团（武宣）100万头生猪产业化项目等重大农业项目建设。

推进现代农业经营体系建设。积极培育新型农业经营主体，力争新增龙头企业5家以上，农民合作社100个以上，家庭农场100个以上。加快知名农产品品牌创建和“三品一标”认证，提升农业品种品质品牌。拓宽农产品新型销售渠道，力争新增农产品电商10家以上。进一步完善农业技术推广、农业机械化、农产品质量安全监测、农业综合执法和农产品流通等服务体系。

（三）大力开展服务业提升年活动，推进现代服务业发展。

以旅游业和商贸物流业为主攻方向，深入实施服务业集聚提升工程，力争全市社会消费品零售总额达157亿元。

大力发展旅游业。扎实推进广西特色旅游名县创建工作，提升金秀旅游名县发展质量，力争忻城县进入创建县、合山市进入备选县行列。加强“圣堂仙境·生态瑶都”和金秀县、象州县“中国长寿之乡”品牌推广。加快推进兴宾蓬莱洲度假区、象州古象温泉度假村二期、忻城土司水街和薰衣草庄园、合山国家矿山公园、武宣庄园文化和革命教育红色旅游等28个重点旅游项目建设。继续抓好一批乡村旅游区和农家乐项目建设。进一步完善旅游公共基础设施。力争实现接待游客1700万人次、旅游收入120亿元以上。

大力发展商贸物流业。加快推进义乌（来宾）小商品批发市场、桂中建材五金家居商贸城、煤炭交易中心、花鸟市场等4个商贸物流项目建设。力争新开工建设桂中汽贸园、铜锣湾商业广场、永合物流园、粮油物流园等项目。积极培育社会消费热点，以会展和节假日消费为重点，支持举办房产、汽车、家居建材、农产品等展销活动。加快平台建设，支持和引导电子商务等新业态发展。

加大限上企业培育力度。促进个体工商户升级为企业，小微企业升级为限额以上企业，力争培育限上企业10家以上。

（四）大力开展项目建设落实年活动，增强经济发展后劲。

统筹推进252个亿元以上重大项目，完成年度投资120亿元以上，力争完成固定资产投资490亿元以上。

抓好西江经济带建设推进年活动。按照“水路和陆路、码头建设和园区建设、工业发展和商贸物流发展、经济发展和生态保护”四个相结合原则，加快西江经济带来宾段建设。实施“港产城一体化”战略，推动沿江城镇发展。利用西江经济带项目滚动增加机制，争取更多项目列入自治区西江经济带基础设施大会战和“双核驱动”战略项目库，加快推进已列入的102个项目建设。

抓好一批项目建设。推进磨东变电站整体迁建工程、凤凰工业园区路网工程等40个重大项目竣工使用。加快来宾水环境综合治理、河南工业园东区集中供热、广西鄂中肥业公司年产60万吨肥料等98个续建项目建设。促进柳州至南宁高速公路（来宾段）改扩建、柳州经合山至南宁高速公路、来宾至凤凰公路、市职业教育中心欧佩克项目等46个重大项目开工建设。着力储备符合融资方向的项目，抓好来宾通用机场、金秀通用机场、兴宾港区莆田作业区一期工程、钟山至来宾高速公路等68个重大项目前期工作。

抓好一批融资项目。积极争取利用国外银行、政府贷款项目及中央预算内资金。充分发挥开发性、政策性金融作用，争取更多资金支持扶贫开发、棚户区改造、水利、公路等领域建设。强化政府引导基金和融资担保体系作用，研究制定促进股权投资和多元融资的扶持政策。进一步探索PPP项目融资新模式，力争实施项目2个以上。

（五）大力开展精准脱贫攻坚年活动，加快贫困群众脱贫致富步伐。

年内减少贫困人口8.5万人以上。突出抓好产业扶贫，对有发展条件和劳动能力的贫困人口实施产业扶贫，增加群众经营性和资产性收入。突出抓好就业扶贫，加强就业技能培训，提高贫困群众的综合素质和谋生技能，有效促进贫困群众就业增收。突出抓好搬迁扶贫，深化拓展新农村建设、城镇化建设、旅游产业、工业园区等易地扶贫搬迁模式，强化配套设施建设和产业项目扶持，实现搬迁扶贫户异地增收发展。突出抓好教育扶贫，切实解决农村家庭困难学生上学难的问题。突出抓好社会救助扶贫，充分发挥政策保障兜底作用，解决一批特殊贫困人口的脱贫问题。同时，深入推进贫困地区路、水、电、网络等基础设施建设。

（六）加强规划建设，推动城乡协调发展。

强化规划引领。推动国民经济和社会发展规划、城乡规划、土地利用规划、生态环境保护规划“多规融合”“多规合一”，强化规划管控。年内实现各县（市）城市控规、乡镇总体规划全覆盖。

推进新型城镇化试点建设。抓好武宣县、象州县和来宾高新区国家新型城镇化深化基础设施投融资体制改革试点、兴宾区自治区农民工创业园建设试点、石龙镇国家建制镇示范试点、桐木镇和红渡镇自治区百镇建设示范工程等建设，围绕解决好农民工进城就业、随迁子女教育、住房、社会福利和保障、户籍和资金筹措等问题，加快推进新型城镇化发展。

提升城区综合承载能力。加快推进教育、医疗、购物、文体活动等基础设施建设，提升城市整体服务能力。加强城区路网建设，完成来宾园博园配套道路、纬十路、中南路延长线等城区道路桥梁建设。加大旧城改造力度，基本完成旧城区道路沥青路面、人行道街砖、雨污管网、绿化美化等方面建设改造。继续推进老汽车站、老火车站等5个片区旧城改造。完善桂中水城项目建设，完成市政渠提升改造，启动维林渠建设，基本实现水城全线贯通。实施桂中大道、滨江公园及来宾北站周边道路景观亮化美化工程。大力推进园博园建设。启动无障碍示范城市建设，完善公共建筑和场所无障碍设施改造。

强化城市服务管理。理顺城市管理执法体制，提高城市管理和公共服务水平。完善市容环境整治长效机制，全面推行数字化城市管理，推动大数据建设，促进城市服务管理向科学化、精细化和现代化发展。

（七）坚持绿色发展，建设美丽来宾。

加强生态环境建设。扎实推进“国家森林城市”“全国生态保护与建设示范区”创建工作，力争完成植树造林20万亩，森林覆盖率达51.75%。深入推进“美丽来宾•生态乡村”活动，建成自治区级绿化示范村屯247个以上、饮水净化示范项目15个以上，打造一批特色美丽乡村。启动建设“宜居乡村”。深入实施石漠化治理、新一轮退耕还林等工程。

强化生态环境保护。加强污水直排口改造、污水处理厂建设，完成污水管网建设31.5公里。实施乡村垃圾治理攻坚行动。推进养殖业技术改造，有效治理养殖污染。加强企业污染物排放监控，严惩违法排污行为。加强备用水源保护和监管。完成自治区下达黄标车和老旧车淘汰任务。

（八）深化改革开放，激发经济发展活力。

推进供给侧结构性改革。充分发挥市场配置资源的决定性作用，提高供给效率。优化农业生产要素和资源，提高农产品供给质量和效率。淘汰落后产能，引导工业产业向中高端迈进，增加工业品有效供给。发挥旅游、生态、健康养生等产业比较优势，提供高品质服务，形成推动经济持续向好发展的新动能。探索以购代建、以租代建、引进民间资本等模式，推进保障性安居工程建设，加大棚户区改造货币化安置力度，消化商品房库存，促进房地产市场健康发展。

深化重点领域改革。深化行政审批制度和商事制度改革。继续推进行政审批规范化管理，抓好行政审批事项调整、承接和下放。完善事中事后监管措施。全面清理规范行政审批前置中介服务事项。推行权力清单、责任清单制度。按照预算法要求，深化部门预算改革。强化财政收入和支出管理。深化农村金融改革，推进“农金村办”。深化国资和国有企业改革，防止国有资产流失。同时，继续抓好农村土地确权、综合行政执法体制、事业单位分类、国有林场、供销社等改革工作。

深化开放与合作。充分利用中国—东盟博览会、商务与投资峰会等平台，加强与东盟国家的交流与合作。积极融入“南柳经济走廊”和“一带一路”战略。

高度重视招商引资。专门抽调精兵强将，组建工业、农业、商贸物流等专门招商工作组，采取专业招商和兼职招商相结合，大力开展产业招商精准招商，招大商、大招商，大力发扬“心容天下，敢为人先”的来宾精神和发挥“天下来宾，来者上宾”的城市对外形象，做到人人关注招商、人人参与招商，强化措施，破除各种制约瓶颈，把招商引资的实际成效与政府部门、县（市、区）政府的年度绩效紧密挂钩，力争在招大引强上实现大突破。

（九）着力改善民生，提高群众获得感。

促进教育振兴发展。深化教育综合改革，提高教育教学质量。抓好教育基础设施建设，加快推进42所从幼儿园到高中的各级各类学校建设，其中市城区建设幼儿园2所、小学10所、初中2所，九年一贯制学校1所，普通高中2所。扩大学前教育资源，学前三年毛入园率达80%。扎实推进义务教育学校标准化建设，确保象州县、武宣县义务教育均衡发展通过国家评估认定，力争兴宾区、金秀县通过自治区评估验收。推进普及高中阶段教育。加快推进市职业教育中心建设发展，加快县级中专综合改革，整体提高职业教育办学水平。大力支持高等教育发展，提高高校服务地方发展的综合能力。

抓好科技文化体育事业发展。实施创新驱动发展战略，加大科技企业孵化器和众创空间建设力度，支持来宾高新区发展高新技术产业。继续巩固和提升“国家公共文化服务体系示范区”“全国全民健身示范城市”“文明城市”创建成果。继续推进国家基层综合性文化服务中心和广西公共体育服务体系试点建设。积极培植文化产业，挖掘打造民族特色文化品牌。实施好重点节庆、主题系列群众文化活动。发展繁荣地方文艺，加强文艺创作，精心打造文艺精品。加强文物保护和非物质文化遗产工作。强化文化执法力度，深入开展“扫黄打非”行动。

推进食品药品、卫生计生事业健康发展。完善食品药品检验检测基础设施。推进“自治区食品安全城市试点市”创建工作。加快推进市妇幼保健院、市全科医生培训基地及一批乡镇卫生院、村卫生室、乡镇医院周转房等卫生计生项目建设。突出抓好疾病预防控制，完善突发公共卫生事件应急体系。加快推进市县乡三级人口健康信息平台建设，逐步实现医疗服务、医疗保障与公共卫生服务信息共享。建立健全新农合统筹工作机制，推进市级新农合数据中心和信息平台建设。实施全面两孩政策。

健全社会保障体系。加大社会保险基金征缴，推进“五险合一”数据大集中和社会保障“一卡通”。完善社会救助体系，健全救灾应急机制。促进“大众创业、万众创新”，加快农民工创业园、创业孵化基地建设，完善创业担保贷款政策，以创业促就业。全年实现城镇新增就业1.3万人以上。农村劳动力新增转移就业2万人次以上。抓好保障房建设。完成农村危房改造9430户。稳步推进大藤峡水利枢纽工程移民安置工作。

抓好为民办实事工程。在完成自治区下达我市为民办实事重点项目的同时，重点实施文化惠民、农村村屯巷道硬化、农民进城就业服务惠民等10项市级为民办实事工程。

深入开展社会治安综合治理和社会矛盾纠纷排查化解，完善灾害事故预防预警和应急处置体系，全面提升人民群众安全感。强化安全生产责任制，遏制重特大安全事故发生。加强国防动员和后备力量建设，努力争创“全国双拥模范城”。依法保障妇女、未成年人和残疾人的合法权益。充分发挥工会、共青团、妇联、文联、红十字会等群团组织的作用。继续做好外事侨务、民族宗教、水库移民、统计、人防、水文、气象、地震、档案等工作。

加强法治政府建设，全面推进依法行政，切实履行政府职能，规范行政权力。优化发展环境，采取有力措施，深入开展“服务工业企业、服务招商引资、服务政务环境”三大专项行动。把各项目标任务落到实处，真正把干部的辛苦指数变成群众的幸福指数。自觉接受人大及其常委会监督，定期向人大及其常委会报告工作，支持人民政协履行政治协商、民主监督、参政议政职能，抓好人大代表议案、建议和政协委员提案办理工作。认真听取各民主党派、工商联、无党派人士和人民团体的意见建议，充分发挥民主协商作用。巩固“三严三实”专题教育成果，严格贯彻落实中央八项规定精神，完善作风建设机制。落实党风廉政建设主体责任和“一岗双责”，推进政府系统党风廉政建设和反腐败工作。

各位代表！为政之道，贵在落实；一分部署，九分落实。我们的蓝图已经绘就，目标已经明确。让我们紧密团结在以习近平同志为总书记的党中央周围，在自治区党委、政府和市委的领导下，紧紧依靠全市各族人民，扎实工作，开拓创新，锐意进取，为“十三五”开好局、起好步，打造广西“双核驱动”战略重要节点城市而努力奋斗！
